# Biogeography of the endosymbiotic dinoflagellates (Symbiodiniaceae) community associated with the brooding coral *Favia gravida* in the Atlantic Ocean

**DOI:** 10.1371/journal.pone.0213519

**Published:** 2019-03-08

**Authors:** Mariana M. Teschima, Amana Garrido, Alexandra Paris, Flavia L. D. Nunes, Carla Zilberberg

**Affiliations:** 1 Programa de Pós-Graduação em Biodiversidade e Biologia Evolutiva, Instituto de Biologia, Universidade Federal do Rio de Janeiro, Rio de Janeiro, RJ, Brazil; 2 Departamento de Biologia Marinha, Instituto de Biologia, Universidade Federal do Rio de Janeiro, Rio de Janeiro, RJ, Brazil; 3 Laboratoire d’Ecologie Benthique Côtière (LEBCO), DYNECO, Ifremer Centre Bretagne, Plouzané, France; 4 Departamento de Zoologia, Instituto de Biologia, Universidade Federal do Rio de Janeiro, Rio de Janeiro, RJ, Brazil; 5 Instituto Coral Vivo, Parque Yayá, Santa Cruz Cabrália, Bahia, Brazil; Newcastle University, UNITED KINGDOM

## Abstract

Zooxanthellate corals live in symbiosis with phototrophic dinoflagellates of the family Symbiodiniaceae, enabling the host coral to dwell in shallow, nutrient-poor marine waters. The South Atlantic Ocean is characterized by low coral diversity with high levels of endemism. However, little is known about coral–dinoflagellate associations in the region. This study examined the diversity of Symbiodiniaceae associated with the scleractinian coral *Favia gravida* across its distributional range using the ITS-2 marker. This brooding coral endemic to the South Atlantic can be found across a wide range of latitudes and longitudes, including the Mid-Atlantic islands. Even though it occurs primarily in shallower environments, *F*. *gravida* is among the few coral species that live in habitats with extreme environmental conditions (high irradiance, temperature, and turbidity) such as very shallow tide pools. In the present study, we show that *F*. *gravida* exhibits some degree of flexibility in its symbiotic association with zooxanthellae across its range. *F*. *gravida* associates predominantly with *Cladocopium* C3 (ITS2 type *Symbiodinium* C3) but also with *Symbiodinium* A3, *Symbiodinium linucheae* (ITS2 type A4), *Cladocopium* C1, *Cladocopium* C130, and *Fugacium* F3. Symbiont diversity varied across biogeographic regions (*Symbiodinium* A3 and *S*. *linucheae* were found in the Tropical Eastern Atlantic, *Cladocopium* C1 in the Mid-Atlantic, and other subtypes in the Southwestern Atlantic) and was affected by local environmental conditions. In addition, Symbiodiniaceae diversity was highest in a southwestern Atlantic oceanic island (Rocas Atoll). Understanding the relationship between corals and their algal symbionts is critical in determining the factors that control the ecological niches of zooxanthellate corals and their symbionts, and identifying host-symbiont pairs that may be more resistant to environmental changes.

## Introduction

The relationship between corals and their intracellular symbiont dinoflagellates (family Symbiodiniaceae) is essential for the survival of shallow water corals and, consequently, the entire coral reef system. Because of this symbiotic relationship, corals can thrive in low-nutrient tropical waters as the endosymbiontics community provides most of the coral host’s metabolic energy requirements, enhancing calcification rates and coral growth [1]. Symbiodiniaceae is a diverse family with different genera, species and strains having different physiologies, photosynthetic efficiencies, and habitat associations [2–4]. Previous phylogenetic studies based on a number of molecular markers have identified nine major ‘*Symbiodinium’* clades assigned alphanumeric nomenclature (A–I) [5], followed by recent taxonomic revision which recognizes the clades as genera and has named them accordingly [6]. Endosymbiontics dinoflagellates are found across a wide range of hosts (e.g., tridacnids, cardiids, sponges, and soft corals [7]), with the genera *Symbiodinium* Hansen & Daugbjerg 2009, *Breviolum* Parkinson & LaJeunesse 2018, *Cladocopium* LaJeunesse & Jeong 2018, *Durusdinium* LaJeunesse 2018, *Fugacium* LaJeunesse 2018, and *Gerakladium* LaJeunesse 2018 (formerly Clades A, B, C, D F and G, respectively) found associating with corals [8]. Symbiodiniaceae have primarily been identified and described using molecular phylogenetics based on the nuclear ribosomal internal transcribed spacer region 2 (ITS2 rDNA) [2,6,9]. Further sampling and advances in Symbiodiniaceae taxonomy has the potential to reveal additional species and new genera.

The association pattern between symbionts and their coral host depends on a variety of factors including, among others, coral species, light exposure, depth, irradiance, host transmission strategy, and symbiont availability in the water column [10]. Many generalist interactions can occur and it is possible for a single colony to harbor different symbiont types [11]. Conversely, specificity between coral host and its symbiont have also been described [12]. The physiological features of each host and symbionts, as well as their composition, are relevant in determining the presence and/or abundance of a specific partner combination [2,11,13]. The diversity of coral-zooxanthellae associations may play an important role in assisting corals to endure environmental changes. Because generation times are shorter for endosymbiotic dinoflagellates than corals, symbionts are likely to adapt more quickly and facilitate the adaptation of corals [4,14]. Moreover, some endosymbionts have been shown to help the host coral acclimate to rising seawater temperatures and survive bleaching events [14–16]. Because temperature anomalies are predicted to occur more frequently and intensely, understanding the diversity of symbionts in various coral hosts is critical to predict the fate of corals under the threat of global climate change.

The diversity of Symbiodiniaceae-coral associations is well documented in the Indo-Pacific and the Caribbean regions. However, little is known about the diversity of host-symbiont associations in corals from the South Atlantic Ocean and how they may vary across environments. *Favia gravida* Verrill, 1868 is a brooding coral that can be found across the South Atlantic Ocean [17]. The species is commonly found in shallow water reefs and tide pools [17] and, together with *Siderastrea stellata* Verrill, 1868, is the most common species found in the shallows. The species is able to survive under extremely harsh conditions including intense solar radiation and high temperatures, although loss of symbionts has been observed during massive coral bleaching events [18]. The distribution of *F*. *gravida* encompasses three marine biogeographic regions: Tropical Southwestern Atlantic (SWA), St Helena and Ascension Islands in the Mid-Atlantic(MA), and the Gulf of Guinea in the Tropical Eastern Atlantic (TEA) [19]. The SWA has a high percentage of endemic coral species [20] and the MA islands, while low in coral richness, can play an important role as stepping stones to reef species crossing the Atlantic [21]. Additionally, the TEA corals do not form true coral reefs as the SWA ones do and their species diversity is low, primarily owing to upwelling events and cooler waters in combination with high volumes of fresh water entering from rivers and heavy rainfall [22]. The Gulf of Guinea is important for its coral fauna and accounts for most of the coral diversity in the TEA, but little is known of its host-symbiont associations [22].

To date, no studies have been published with respect to Symbiodiniaceae from the Gulf of Guinea. In the MA, symbiont diversity have been described associated with zoanthids in Ascension Island [23] and with a sea anemone in St Helena Island [24]. Along the SWA coast, some biogeographical barriers seem to exist for Symbiodiniaceae species and the diversity of three different genera is partitioned by environmental variables: *Symbiodinium* spp. correlates with high irradiance, *Breviolum* spp. with low temperatures, and *Cladocopium* spp. with high turbidity [25]. To our knowledge, only two other studies have examined Symbiodiniaceae–coral associations in the SWA, but they were each restricted to two sampling sites. *Siderastrea* sp. from northeast Brazil associated with different lineages, mostly from *Cladocopium* spp. (C1, C3, and C46) but also with *Breviolum* B5 [26], whereas *Mussismilia* spp. Ortman, 1890 associated with *Symbiodinium linucheae* (Trench & Thinh) LaJeunesse, 2001 (ITS2 type A4), *Breviolum* B19, and *Cladocopium* C3 [27]. Different host taxa including zoanthids and sea anemone from the SWA coast have also been found associated with *Symbiodinium microadriaticum* Freudenthal, 1962 (ITS2 type A1), *S*. *linucheae*, *Breviolum* B1 and B2, and *Cladocopium* C1 [24,28,29]. Even though the association between the sister species *Favia fragum* (Esper, 1795) and its algal symbionts has been examined in the Caribbean [10], no studies have investigated the endosymbionts diversity and distribution in *F*. *gravida*.

In this study, we characterized the diversity of the endosymbiotic dinoflagellates associated with the scleractinian coral *F*. *gravida* across its distributional range in the South Atlantic. We examined (i) whether any specificity was observed in the *F*. *gravida*-symbiont association; (ii) whether new endosymbionts lineages were identified in the South Atlantic; (iii) whether *F*. *gravida* harbored different Symbiodiniaceae communities across its range.

## Materials and methods

### Sampling sites and collection

Samples of *Favia gravida* (< 1 cm²) corals were collected between February 2004 and July 2017, despite the difference between collected years no anomalies (e.g. higher water temperature or coral bleaching) were recorded during sampling. Collection encompass 12 locations in the South Atlantic Ocean (S1 File), specifically in the SWA: Ceará (CE), Rocas Atoll (RA), Fernando de Noronha Island (FN), Rio Grande do Norte (RN), Paraíba (PB), Pernambuco (PE), Alagoas (AL), Porto Seguro (PS), Abrolhos (AB), Trindade Island (TR); in the MA: Ascension Island (ASC); and in the TEA: São Tomé Island (ST). Permission to collect coral samples was approved by IBAMA (permits 22387–2, 02001.005656/2005-57, 968–1, 29953–2, 29687–4), Secretaria Municipal de Meio Ambiente de Porto Seguro (10/2015), and IDEMA (24/2016) in Brazil, Direcção das Pescas in São Tomé & Príncipe (CITES 139/DP/ME/05) and Conservation Department, Ascension Island Government (CITES 01/16). Small fragments of colonies were collected by scuba diving or snorkeling using a hammer and chisel. The number of colonies sampled per site ranged from 5 to 35 depending on the coral’s abundance at each site (Table 1). Samples were stored in CHAOS solution (4 M guanidine thiocyanate, 0.1% N-lauroylsarcosine sodium, 10 mM Tris pH 8.0, 0.1 M 2-mercaptoethanol) at 4°C until analyzed.

**Table 1:** Summary of samples information: All sampled sites, number of samples per site, endosymbionts identified, depth, date, season of sampling, and GenBank code.

Site	N sampled	Symbiodiniaceae			Depth	Collected date	GenBank
São Tomé (ST),	19	*S*. A3	*S*. *linucheae*		tide pool and up to 2 m	Feb., 2006	MH931833 –
Tropical Eastern Atlantic (TEA)		N = 4	N = 7			Rainy season	MH931843
Ascension Island (ASC),	14	*C*. C1			tide pool	Oct., 2013	MH931886 –
Mid-Atlantic (MA)		N = 10				Dry season	MH931895
Ceará (CE),	12	*C*. C3			4 m	Jul., 2017	MH931936 –
Southwestern Atlantic (SWA)		N = 10				Dry season	MH931945
Rio Grande do Norte (RN),	13	*C*. C3			1.5 m	Out., 2016	MH931896 –
Southwestern Atlantic (SWA)		N = 6				Dry season	MH931900
Rocas Atoll (RA),	32	*S*.*linucheae*	*C*. C1		tide pool and up to 4 m	May, 2014	MH931830
Southwestern Atlantic (SWA)		N = 4	N = 2			May, 2017	MH931831
						Rainy season	MH931845
		*C*. C3	*F*. F3				MH931846
		N = 5	N = 1				MH931879 –
							MH931885
							MH932002
Fernando de Noronha (FN),	34	*S*.*linucheae*	*C*. C3		tide pool and up to 13 m	Set., 2014	MH931847
Southwestern Atlantic (SWA)		N = 2	N = 24			Oct., 2016	MH931848
						Dry season	MH931910 –
							MH931935
Paraíba (PB),	22	*S*.*linucheae*	*C*. C1	*C*. C130	2 m	Jan., 2004	MH931849 –
Southwestern Atlantic (SWA)		N = 6	N = 5	N = 3		Dry season	MH931854
							MH931946 –
							MH931953
Pernambuco (PE),	13	*S*.*linucheae*	*C*. C1	*C*. C3	tide pool	Apr., 2016	MH931855
Southwestern Atlantic (SWA)		N = 1	N = 1	N = 3		Rainy season	MH931954 –
							MH93195
Alagoas (AL),	5	*S*.*linucheae*			tide pool	Jun., 2016	MH931832
Southwestern Atlantic (SWA)		N = 2				Rainy season	MH931844
Porto Seguro (PS),	45	*C*. C3			2 m	Oct., 2015	MH931958 –
Southwestern Atlantic (SWA)		N = 31				Dry season	MH931988
Abrolhos (AB),	34	*C*. C3			4 to 9 m	Nov., 2014	MH931857 –
Southwestern Atlantic (SWA)		N = 22				Rainy season	MH931878
Trindade Island (TR),	35	*S*.*linucheae*	*C*. C1	*C*. C3	tide pool and up to 8 m	Jun., 2012	MH931856
Southwestern Atlantic (SWA)		N = 1	N = 17	N = 5		Sept., 2014	MH931901 –
						Rainy season	MH931909
							MH931989 –
							MH932001

### DNA extraction, 28S RFLP and ITS2 sequencing

DNA was extracted with phenol-chloroform and ethanol precipitation as described by Fukami et al. [30] and resuspended in Milli-Q water with RNase A (10 mg/ml; Sigma-Aldrich, St. Louis, MO, USA).

To check for the absence of Symbiodiniaceae genera mixture, screening by restriction fragment length polymorphism (RFLP) was performed by amplifying the large subunit ribosomal gene (28S rDNA), using the primers D1D2 Zoox F (5’–CCT CAG TAA TGG CGA ATG AAC A–3’) and D1D2 Zoox R (5’–CCT TGG TCC GTG TTT CAA GA–3’) [31], under the following conditions: 5 min at 94°C followed by a pre-cycle of 1 min at 94°C, 1 min at 56°C and 45 s at 72°C, and 29 cycles of 1 min at 92°C, 1 min at 56°C and 45 s at 72°C followed by 10 min at 72°C. Restriction digestions were performed by incubating 3 μL of the amplified product with 10 U of restriction enzyme *Taq* I (Thermo Fisher Scientific, Waltham, MA, USA) at 65°C overnight. Digested fragments were separated by electrophoresis in a 2% agarose gel, visualized under UV light, and compared with digest patterns from monoclonal cultures of *Symbiodinium* sp., *Breviolum* sp., *Cladocopium* sp. and *Durusdinium* sp., kindly donated by Dr. Mark Warner (University of Delaware, DE, USA). If a single clade were found in a sample, the ribosomal internal transcript spacer 2 region (ITS2 rDNA) was amplified, using the ITSint-for2 (5’–GAA TTG CAG AAC TCC GTG–3’) [32] and ITS2-rev (5’–GGG ATC CAT ATG CTT AAG TTC AGC GGG T–3’) primers [33], and sequenced. PCR conditions followed those of LaJeunesse and Trench [32] with a touchdown amplification protocol, starting with: 5 min at 94°C, 45 s at 94°C, 45 s at 62–52°C (starting at 62°C and decreasing one degree every two cycles until 52°C), and 30 s at 72°C. Next, the reaction continued for an additional 20 cycles of 45 s at 94°C, 45 s at 52°C, 30 s at 72°C, and a final extension of 10 min at 72°C. The PCR products were visualized by 1% agarose gel electrophoresis.

Sequencing was performed on successfully amplified and purified PCR products in the forward direction using an ABI 3500 genetic analyzer with the BigDye Terminator v3.1 kit (Applied Biosystems, Foster City, CA, USA). Samples with more than one clade on RFLP screening or with additional unidentified bands were cloned using the CloneJET PCR Cloning Kit (Thermo Scientific, Waltham, MA, USA) with competent *E*. *coli* DH5α transformed using TransformAid Bacterial Transformation Kit (Thermo Scientific) following the manufacturer’s protocol. Each transformed sample was plated in two Luria-Bertani (LB) agar plates and kept at 37°C overnight for the growth of bacterial colonies. At least 48 clones were isolated from the growth plates and stored at −80°C in a clone library arrayed on a 96-well plate with 200 μl of liquid LB medium with 30% glycerol. Amplification reactions and sequencing of at least 16 clones from each sample using the ITS2 rDNA primers were performed as described above to identify dominant Symbiodiniaceae lineages. Only dominant haplotypes identified by bacterial cloning were included in the downstream analyses to avoid an overestimation of biodiversity due to cloning artifacts and/or intragenomic ITS2 variation [34,35].

### DNA sequencing and analysis

Sequence chromatographs were manually checked and edited using the Geneious R11 software (Biomatters, Auckland, New Zealand). To ascertain that recovered sequences were from Symbiodiniaceae and to determine which genera they belonged to, BLAST (Basic Local Alignment Search Tool) searches were performed in the National Center for Biotechnology Information (NCBI) database. When a Symbiodiniaceae genera or species was identified in our samples (BLAST parameters: query cover 99–100%, E value ≤ 0.01, identity ≥ 98%), sequences from that particular species/lineage were downloaded from the NCBI database and aligned with the ones from this study using ClustalW alignment in Mega 7 software [36] for the phylogenetic and phylogeographic analyses. Samples number decreased from collection to identification because of unsuccessful results during the process (PCR failure and unclear sequencing electropherograms).

For *Cladocopium* spp., ITS2 sequences were obtained from GenBank for C1, C3, and C130, which were found to be similar to our sequences run through BLAST. *Cladocopium* C81, which has been found in association with the sister host species *F*. *fragum*, was chosen as the outgroup. For *Symbiodinium spp*., sequences for *Symbiodinium* A3 and *S*. *linucheae* were obtained from GenBank and *S*. *microadriaticum* was used as the outgroup. Lastly, GenBank sequences of *Fugacium* F3 were used for phylogenetic analysis with *F*. *kawaguttii* Trench & Blank, 2000 (ITS2 type F1) as the outgroup. Bayesian and maximum-likelihood phylogenetic trees were reconstructed separately for each clade using MrBayes 3.2.6 software [37] and PhyML platform [38], respectively. The best model of sequence evolution for the dataset was determined by the AIC criterion using jModelTest 2 [39]. The Bayesian analysis was run for two million generations and sampled every 1,000 generations with four chains, the minimum chain length required for the average standard deviation of split frequencies to reach a value of 0.01, and a burn-in of 0.25. The heuristic search option for the maximum-likelihood trees was subtree pruning and regrafting with 1,000 bootstrap replicates. The phylogenetic tree for each Symbiodiniaceae genera was edited using FigTree 1.3.1 [40]. For the phylogeographic analysis, haplotype networks were constructed for each genera using the median-joining method in PopArt software [41,42]. Identical haplotypes were excluded from phylogenetic analyses but were kept for haplotype networks. Sequences from GenBank of the same ITS2 lineage found in this study were included in the haplotype network to compare their geographic distributions.

## Results

Most RFLP analyses followed a pattern found for major Symbiodiniaceae genera by Baker and Rowan [43]. In addition, sequencing data supported the RFLP results, which indicate that RFLP was a good general predictor for dominant endosymbionts in this study. Only four sites (RA, FN, RN, and PS) had samples with unclear RFLP patterns for which cloning was required. Bacterial cloning of PCR products revealed that, in most cases, RFLP patterns that did not clearly match an expected single-clade pattern were caused by incomplete digestion by the restriction enzyme. Nearly all corals examined harbored a single symbiont type. Only coral samples from the RA had mixtures of different Symbiodiniaceae genera in the same colony (one colony each hosted *S*. *linucheae*/*Fugacium* F3 and *S*. *linucheae*/*Cladocopium* C1, respectively).

**Fig 1:**
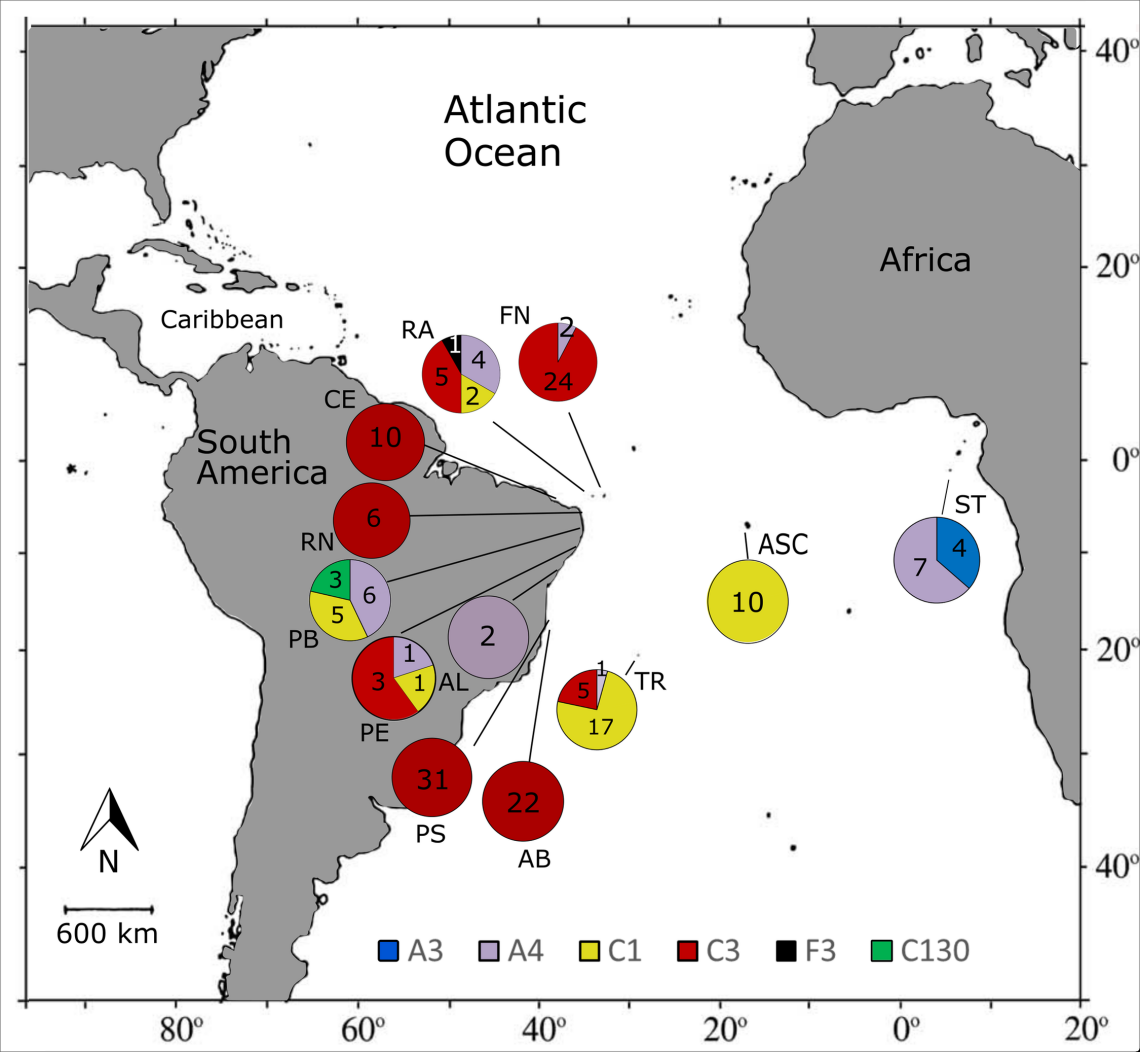
Sampling sites and geographic distribution of dominant endosymbiotic dinoflagellates associated with *Favia gravida* in the Southern Atlantic Ocean. Numerals inside the circles indicate the number of colonies found associated with the respective endosymbiont lineages. Tropical Eastern Atlantic: ST = São Tomé; Mid-Atlantic: ASC = Ascension Island; Southwestern Atlantic: CE = Ceará, RA = Rocas Atoll, FN = Fernando de Noronha, RN = Rio Grande do Norte, PB = Paraíba, PE = Pernambuco, AL = Alagoas, PS = Porto Seguro (Bahia), AB = Abrolhos (Bahia), and TR = Trindade Island.

*Cladocopium* C3 was the most prevalent species (61.6%, N = 106) found in association with *F*. *gravida* (Fig 1). This endosymbiont was identified in coral samples collected from eight of 12 sampling sites, all in the SWA. *Cladocopium* C1 was identified in samples from the SWA and MA and was the second most common subtype, accounting for 20.3% (N = 35) of all samples. *S*. *linucheae* was identified in coral samples from the SWA and TEA and was the third most dominant subtype associated with *F*. *gravida* (13.4%, N = 23). The other endosymbionts identified in this study (*Symbiodinium* A3, *Cladocopium* C130, and *Fugacium* F3) combined accounted for less than 5% (N = 7) of all coral samples.

**Fig 2:**
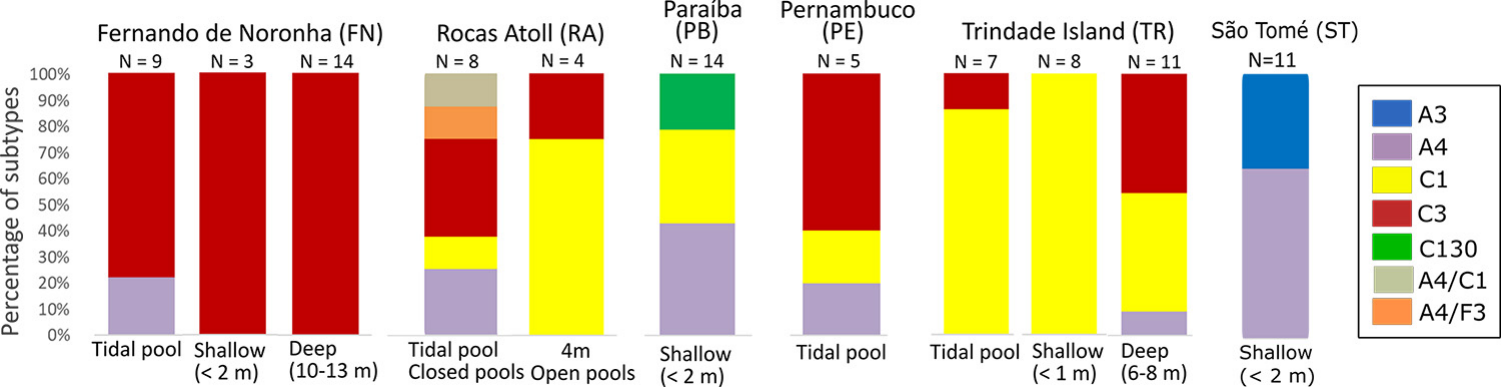
Proportion of each Symbiodiniaceae lineages per depth at sampling sites with more than one subtype.

Coral samples from the MA region harbored exclusively lineage *Cladocopium* C1, whereas colonies collected from the TEA only hosted *Symbiodinium* A3 and *S*. *linucheae*. Symbiodiniaceae diversity was highest in the SWA: five lineages (*S*. *linucheae*, *Cladocopium* C1, *Cladocopium* C3, *Cladocopium* C130, and *Fugacium* F3) were found either alone in one coral colony or two lineages combined (Figs 1 and 2). Even though the SWA covers a larger latitudinal range, encompassing most of the distributional range of *F*. *gravida* on this side of the Atlantic, one-half of the sampling sites had only one dominant Symbiodiniaceae lineages. Multiple dominant endosymbionts were found in two of seven coastal sites and all three oceanic islands in Brazil. In addition, *Cladocopium* C3 was not identified in corals from only two coastal sites in the SWA (PB and AL). As mentioned above, corals harboring mixed endosymbionts lineages were only found in the RA. Symbiodiniaceae diversity was highest in the RA where *Cladocopium* C3 and *S*. *linucheae* were dominant, accounting for 75% (N = 9) of samples, whereas *Fugacium* F3 was the least abundant, found only in one sample co-occurring with *S*. *linucheae*.

The lineages *Cladocopium* C1, *Cladocopium* C3, and *S*. *linucheae* were the most abundant in the six sampling sites where more than one endosymbiont lineage was identified (Fig 2). Six different endosymbionts lineages were found in reefs from 0–2 m habitats while only three were found in reefs from 4–13 m sites. *Cladocopium* C3 was the only subtype identified in samples from CE, AB, RN, and PS, despite larger sample sizes in AB (N = 22) and PS (N = 31) (Fig 1). Additionally, either *Cladocopium* C1 and/or *Cladocopium* C3 were identified in corals collected from all sites except ST and AL. AL was undersampled and more data would be needed to confirm this pattern. *Cladocopium* C130 was only identified from corals collected from PB.

**Fig 3:**
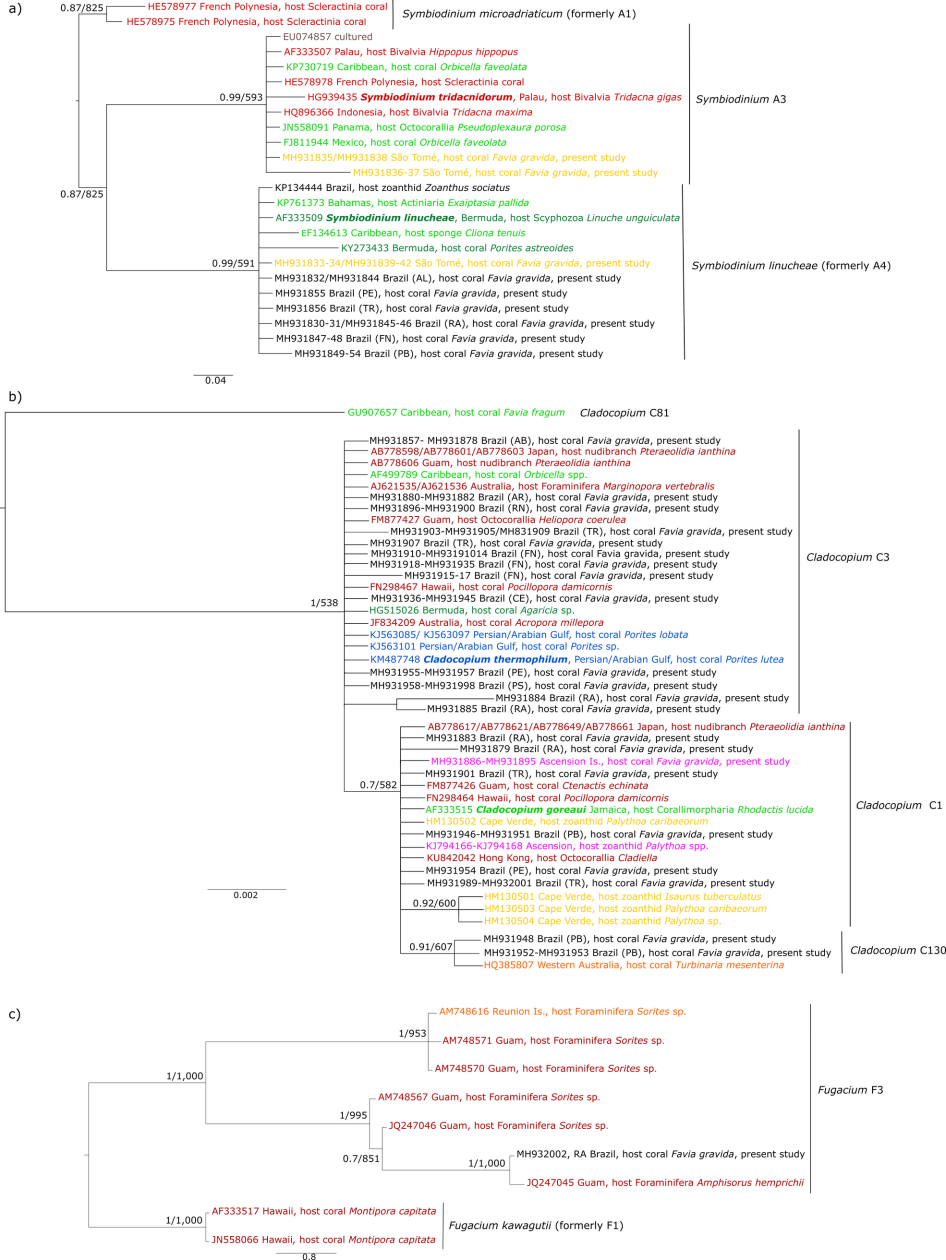
Bayesian phylogenetic tree based on Symbiodiniaceae ITS2 sequences of *Cladocopium* spp. **(a), *Symbiodinium* spp. (b), and *Fugacium* spp. (c)**. Posterior probability distributions and bootstrap values of 1,000 replicates from maximum-likelihood analysis are shown for branch support. GenBank accession numbers are indicated on the tree. Sampling site abbreviations from this study are explained in Fig 1. Colors represent biogeographic regions: dark green = Northwestern Atlantic, light green = Caribbean, black = Southwestern Atlantic, pink = Mid-Atlantic, yellow = Tropical Eastern Atlantic, blue = Red Sea, red = Pacific Ocean, and orange = Indian Ocean.

The phylogenetic trees of *Symbiodinium* spp. and *Cladocopium* spp. (Fig 3A and 3B) support the identification of *Symbiodinium* A3, *S*. *linucheae*, *Cladocopium* C1, *Cladocopium* C3, and *Cladocopium* C130. The KP134444 sequence from a *Symbiodinium* sp. hosted by zoanthids in Brazil previously identified as belonging to *Symbiodinium* A3 by Rabelo et al. [29] clustered with *S*. *linucheae* sequences in our analysis (Fig 3A). Even though *Symbiodinium* A3 sequences grouped with *S*. *tridacnidorum* Lee, Jeong, Kang & LaJeunesse, 2015 (ITS2 type A3 from the Pacific) we decided to keep it as *Symbiodinium* A3 because there is a discussion about *Symbiodinium* A3 from the Atlantic being a different species [6]. Similarly, all *Cladocopium* C1 sequences included in our analyses clustered with *C*. *goreaui* (Trench & Blank, 2000) and *Cladocopium* C3 sequences clustered with *C*. *thermophilum*, (Hume, D’Angelo, Smith, Stevens, Burt & Wiedenmann, 2015) the only *Cladocopium* species described for now. However *Cladocopium* is the most diverse genus in Symbiodiniaceae and distinct species may yet be described within the group (ITS2 types) [44]. Given current taxonomic uncertainty within the group, we maintain a conservative assignment at the genus level. Symbionts from the South Atlantic identified as *Fugacium* grouped with *Fugacium* F3 from GenBank. Furthermore, phylogenetic analysis of published sequences and data from this study show more than one well supported clades within *Fugacium* F3 found across all hosts (Fig 3C).

**Fig 4:**
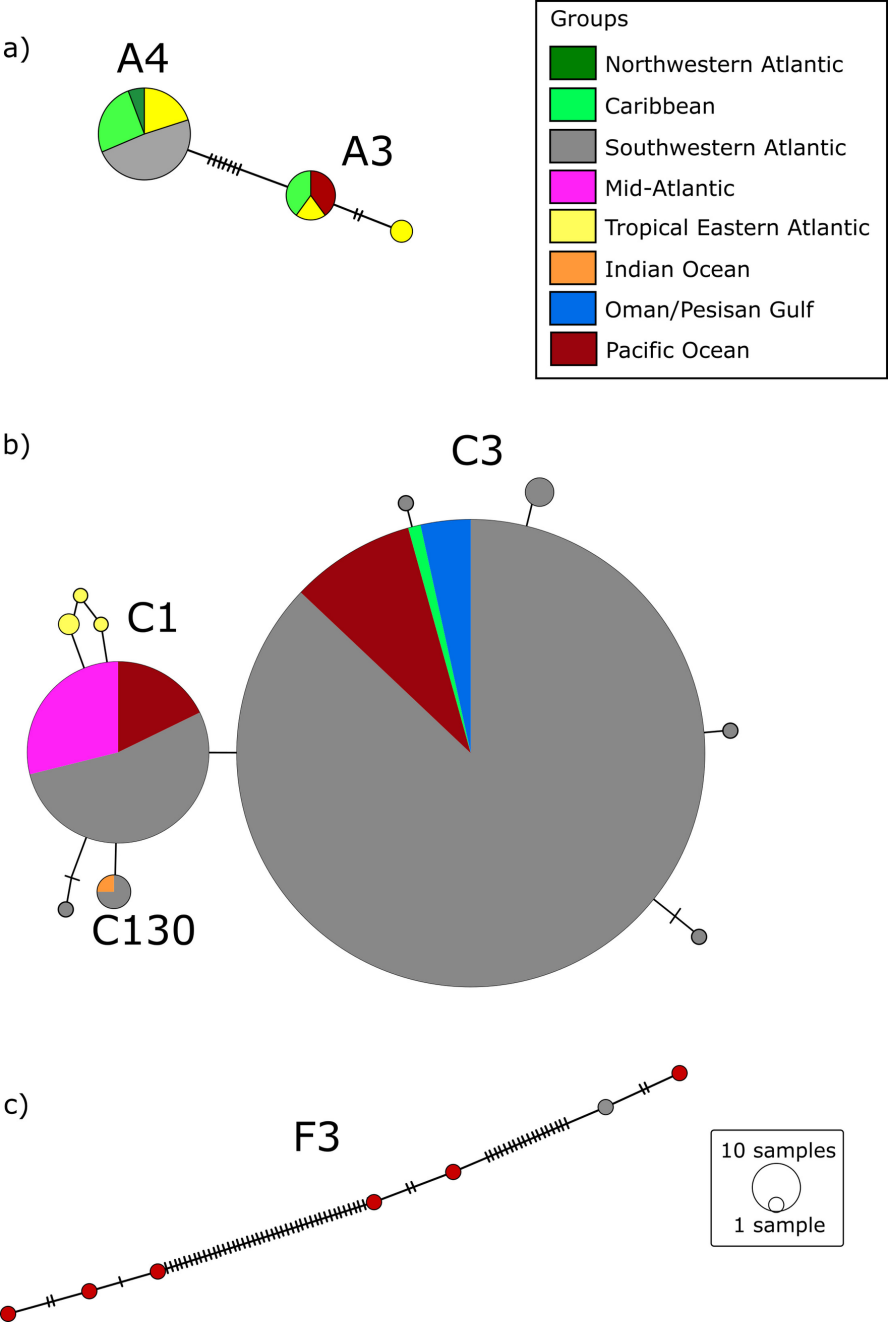
Median-joining haplotype network of endosymbiotic dinoflagellates *Symbiodinium* spp. **(a), *Cladocopium* spp. (b) and *Fugacium* spp. (c) with sequences from this study and from the GenBank.** Black dashes correspond to mutational events.

The *Symbiodinium* spp. haplotype network showed that *F*. *gravida* samples collected in our study from the TEA and SWA shared the same *S*. *linucheae* haplotype, which has also been identified in samples from the Northwestern Atlantic and the Caribbean (Fig 4A). As mentioned before, *Symbiodinium* A3, within our dataset, was only identified in samples from ST. For this lineage one haplotype is exclusive to our ST samples and another is in common to the Caribbean and the Pacific Ocean (Fig 4B). *Cladocopium* C1 and C3 haplotypes differ by a single nucleotide (Fig 4B). The most common *Cladocopium* C1 haplotype found in this study (SWA and MA) has been previously identified in samples collected from the Pacific Ocean but it differed from the ones found in zoanthids in the TEA. Additionally, SWA had an exclusive *Cladocopium* C1 haplotype and the *Cladocopium* C130 haplotype is common to the SWA and the Indian Ocean. In the case of the *Cladocopium* C3 (SWA) identified in this study the most common haplotype is identical to samples retrieved from GenBank from the Caribbean, the Oman and Persian Gulfs, and the Pacific Ocean, with the exception of a few haplotypes variants from the oceanic islands of the SWA (FN, RA and TR), which differed from the most common haplotype by one or two bp mutation. Although, *Fugacium* F3 sequences were more similar to each other than to another *Fugacium* group, all haplotypes, including ours from the SWA and GenBank sequences from the Pacific were different from one another (Fig 4C).

## Discussion

### General patterns in the association between Symbiodiniaceae and *Favia gravida*

In this study, we demonstrate that the scleractinian coral *Favia gravida* is a generalist host that may harbor Symbiodiniaceae lineages from three different genera and six lineages (*Symbiodinium* A3 and *S*. *linucheae*, *Cladocopium* C1, C3 and C130, and *Fugacium* F3). While no new lineages of Symbiodiniaceae were identified, we report on the first occurrence of *Cladocopium* C130 and *Fugacium* F3 in the South Atlantic. *F*. *gravida* had a stronger association with *Cladocopium* C3, which was found in nearly 70% of sampling sites, some over 2,200 km apart. *Cladocopium* C3 is a widely distributed generalist symbiont that has been found in association with a variety of coral species [12,44]. It has been suggested that *Cladocopium* C1 and *Cladocopium* C3 may be ancestral stock species within *Cladocopium* from which other types have been derived [44], which could explain their wide geographical range and association with a wide variety of hosts.

Symbiodiniaceae associations with *F*. *gravida* have a discernable biogeographical pattern. The TEA host populations harbor only *Symbiodinium* and the MA populations harbor only *Cladocopium*. The SWA corals harbor the highest endosymbionts diversity (*Symbiodinium*, *Cladocopium* and *Fugacium*), with *Cladocopium* C3 being the most common association. Interestingly, while the sister species *F*. *fragum* from the Caribbean associates mostly with *Breviolum* spp.[10], this symbiont genus was not found in the current study despite intensive sampling. A large amount of *Breviolum* spp. can be found in the Caribbean as a result of an ecoevolutionary expansion of this genus during Pliocene-Pleistocene [45] probably the physical-environment conditions that propitiated these endosymbionts radiation was not the same in the South Western Atlantic where *Breviolum* spp. occur but do not seem to stand out.

### Symbiodiniaceae diversity by biogeographic region

To our knowledge, this is the first study of coral symbiont algal diversity in the Gulf of Guinea, TEA. At the ITS2 lineage level, the TEA had a higher diversity of *Symbiodinium* spp. (*Symbiodinium* A3 and *S*. *linucheae*) compared to the SWA (*S*. *linucheae*). These two lineages have previously been identified in coral samples collected from the Atlantic, where *Symbiodinium* spp. seems to be more common relative to the Pacific Ocean [46]. Studies in the Northeastern Atlantic (Madeira Island, Canary Islands, and Cape Verde) with different host groups (corals, sea anemone, and zoanthids) revealed a greater diversity of Symbionidiaceae (*Symbiodinium* sp., *Breviolum* sp., *Cladocopium* sp. *and Durusdinium* sp.) [47] than the current study, and more specifically the lineages *Breviolum* B1 [24], *Cladocopium* C1, *Cladocopium* C3, *Cladocopium* C21 [48], and *Cladocopium* C46 [26]. Even though *F*. *gravida* is a generalist host, it was only found associated with the genus *Symbiodinium* in the Gulf of Guinea. Additional studies with other reef-building corals and host taxa in the TEA, particularly in São Tomé, are needed to further characterize the Symbiodiniaceae diversity of this region.

Endosymbionts diversity was the lowest in the MA, where only *Cladocopium* C1 was identified compared to three lineages of *Cladocopium* found in the SWA (*Cladocopium* C1, *Cladocopium* C3, and *Cladocopium* C130). Two studies from the MA showed a low diversity of symbionts, namely one *Cladocopium* C1 and *Breviolum* B1 each associated with zoanthids [23] and a sea anemone [24], respectively. In our study, *F*. *gravida* samples from the MA were collected in semi-enclosed tide pools infrequently connected to the sea and possibly exposed to air at low tide [17]. Thus, salinity, temperature, and other environmental parameters in those sites must be more extreme than subtidally, and *Cladocopium* C1 may be able to tolerate a wide range of conditions perhaps by the presence of protective compounds such as UV-blockers [49], even though we expected to find members of *Symbiodinium* in ASC as observed in other shallow sites. Juveniles of the common Indo–Pacific coral *Acropora tenuis* (Dana, 1846) were found to have much greater thermal tolerance when associated with *Cladocopium* C1 [50]. Moreover, in Australia *Cladocopium* C1 occurs in shallower, high-irradiance habitats and was found to be substantially more heat tolerant than *Cladocopium* C3 [44]. Alternatively, because ASC is a remote oceanic island, symbiont diversity may be intrinsically low. In fact, a report on the Zoantharia fauna of ASC found that two zoantharian species, *Palythoa caribaeorum* (Duchassaing & Michelotti, 1860) and *Palythoa* aff. *clavata* (Duchassaing, 1850), were in association exclusively with only one symbiont species, *Cladocopium* C1 [23]. Again, the MA is a poorly sampled area and additional studies at a broader scale with a larger number of taxa are needed to characterize the existing Symbiodiniaceae diversity of the MA islands.

In the SWA, a variety of endosymbionts lineages have been identified associated with corals, zoanthids, and sea anemone (e.g., *Symbiodinium* A3, *S*. *linucheae*, *Breviolum* B1, *Breviolum* B5, *Breviolum* B19, *Cladocopium* C1, *Cladocopium* C3, *Cladocopium* C46; [24,26–29]). In the current study, *F*. *gravida* colonies collected from the SWA were found associated with subtypes *S*. *linucheae*, *Cladocopium* C1, and *Cladocopium* C3, in addition to two new occurrences, *Cladocopium* C130 and *Fugacium* F3. Also, we believe that the *Symbiodinium* previously identified as *Symbiodinium* A3 by Rabelo et al. [29] was misidentified. The phylogenetic analysis presented in Rabelo et al. [29] only included *Symbiodinium* A3. However, when sequences from other *Symbiodinium* lineages are included, as presented here, phylogenetic analysis of all SWA sequences formed a single cluster with *S*. *linucheae* (including the sequence from Rabelo et al. [29]). Thus, current available data and analyses do not support the presence of S*ymbiodinium* A3 in the SWA.

Higher symbiont diversity found in the SWA compared to the TEA and MA could be in part explained by the higher sampling effort on the SWA. Nevertheless, greater symbiont diversity is observed in the oceanic islands of SWA compared to the TEA and MA, supporting the interpretation of greater symbiont diversity in the SWA. In fact, coral populations from RA harbored the highest symbiont diversity in our study, represented by three Symbiodiniaceae genera and four lineages. Similar results were reported for genus *Siderastrea* Blainville, 1830 symbionts sampled in eastern compared to western Atlantic [26]: *Siderastrea* spp. from Cape Verde (TEA) harbored only one endosymbiont lineages, whereas up to five lineages were found in Northwestern Atlantic (NWA) corals. Coral species in the genus *Oculina* Lamarck, 1816 from the NWA also harbored greater symbiont diversity in a study that surveyed Symbiodiniaceae communities from the Caribbean and the Mediterranean [51]. Thus, the Western Atlantic appears to possess a higher diversity of symbionts compared to the TEA and MA, and possibly the Mediterranean as well. The greater symbiont diversity of the Western Atlantic may be explained by the previous connectivity between the Atlantic and Pacific prior to the closure of the Isthmus of Panama, the shifts in climate conditions that favored the diversification of Symbiodiniaceae following this event during the Pliocene, and its greater diversity and availability of habitats [44,45].

Biogeography of marine organisms in the South Atlantic shows a general trend of greater diversity in the SWA. However, patterns of species diversity in the TEA and the MA varies according to taxonomic group, such that for some groups of organisms, there is greater similarity between MA and SWA (e.g. reef fishes [52] and zoantharians [23]) and for others, that MA is more similar to the TEA (e.g reef fishes [21] and marine invertebrates [53]). According to our observations for Symbiodiniaceae, MA shares one symbiont lineage with SWA and none with TEA. In this case, MA is more similar to SWA than TEA. Conversely, a global study of the sea anemone *Exaiptasia pallida* (Agassiz in Verrill, 1864) [24] showed that it harbored *Breviolum* B1 in Brazil, Canary, Madeira and St Helena islands, indicating that the endosymbiotic dinoflagellates are able to disperse across the Atlantic. Thus, specific local conditions may impact the host-symbiont association but not the dispersal ability of Symbiodiniaceae in the Atlantic.

### Symbiodiniaceae distribution in the Southwestern Atlantic (SWA)

Corals often show preference for a particular symbiont type but new combinations can be formed in response to changes in local physical environmental conditions [54,55]. In the SWA, *Cladocopium* C3 (the most prevalent in the region) and *S*. *linucheae* were found concomitantly in most sites comprised by a variety of habitats. However, *S*. *linucheae* was more prevalent in tide pools or shallow reefs than deeper reefs. *Symbiodinium* spp. is known to synthesize mycosporine-like amino acids (MAAs), which naturally absorb UVA radiation [56] thereby protecting the holobiont symbionts in high-irradiance habitats. Additionally, *Symbiodinium* spp. can assimilate more inorganic nitrogen than members of *Cladocopium* spp. and increase the metabolic activity of their hosts [57]. An alternative explanation for the higher prevalence of *S*. *linucheae* in shallow waters is that members of *Symbiodinium* spp. are opportunistic or parasitic symbionts [8]. High temperatures can be harmful to corals [14], tide pool corals exposed to acute thermal stress may become more susceptible to bleaching or disease, resulting in the proliferation of opportunistic symbionts in health-compromised corals.

Three Symbiodiniaceae genera have been identified in association with the endemic reef-building coral *Mussismilia hispida* (Verrill, 1901) along the SWA coast [25], interestingly, the endosymbiont distributional pattern was very different from that of *F*. *gravida*, even for samples collected at the same site. Even though the two species can co-occur, they usually occupy different habitats in the reef: *F*. *gravida* corals are found preferentially in very shallow waters up to 5 m, especially in tide pools, whereas *M*. *hispida* is often found below 3‒5 m. Additionally, the endosymbionts associated with *M*. *hispida* were found to clearly exhibit a latitudinal gradient that correlates with temperature and turbidity and closely follows the distribution of reef corals in the SWA, but a similar pattern was not found for the endosymbionts in *F*. *gravida*. While only members of *Symbiodinium* were found associated with *M*. *hispida* in the northeast coast of Brazil [26], five lineages from three different genera (*Symbiodinium*, *Cladocopium*, and *Fugacium*) were identified in *F*. *gravida* samples in our study. Similarly, symbiont diversity in *Siderastrea* spp. from PB on the northeast coast of Brazil was higher than in most sites in the Caribbean [26]. In the current study, PB exhibited high symbiont diversity and one lineage (*Cladocopium* C130) was found exclusively at this site. In fact, this coastal site was among the richest in terms of endosymbionts diversity associated with *F*. *gravida* (this study) and *Siderastrea* spp. [26] with three and four lineages being observed, respectively. Host-symbiont associations in the northeast coast of Brazil may be influenced by environmental conditions, host specificity, ecological parameters, and a combination of these factors and further studies are needed to address their relationships.

To date, only *Cladocopium* spp. (*F*. *gravida*, *Cladocopium* C3; *Siderastrea* spp., *Cladocopium* C1 and *Cladocopium* C46; *M*. *hispida*, *Cladocopium* sp.) have been observed in Bahia, northeast Brazil (PS and AB in this study). The coast of Bahia receives large amounts of terrigenous sediments (40‒80%) and the Abrolhos Bank, in particular, is described as having sedimentation rates up to 10 mg cm^−^² day^−^^1^, which is the maximum estimated limit for corals which are not subject to stresses from human activities [58]. This factor could explain the predominance of *Cladocopium* genus in Bahia, since the presence of *Cladocopium* C3 symbionts is often observed in environments with lower irradiance level [44] and correlated with turbidity in the SWA [25]. Although *S*. *linucheae* was previously recorded in the region in association with *Mussismilia braziliensis* (Verrill, 1868) [27], the coral sample was collected in the outer reefs of Abrolhos, three times further from the coast than PS, and where waters are likely to be less turbid. In Rio Grande do Norte (RN), where corals are also highly affected by terrestrial sediments [20], *Cladocopium* C3 was the only endosymbiont found in samples of *F*. *gravida*. Another possible explanation for the predominance of *Cladocopium* C3 in the northeast coast of Brazil is the development of a host-specialist *Cladocopium* C3 lineage for *F*. *gravida* as it occurred in other Caribbean corals hosts species [44]. A phylogenetic study using a highly variable marker (e.g., psbA) is needed to verify the existence of other lineages of *Cladocopium* genus.

Finally, special attention should be given to the SWA oceanic islands because, unlikely ASC and ST, they showed higher symbiont diversity. One possible explanation for finding more than one lineage of endosymbionts at SWA islands is that these islands served as refuge during low sea-level periods and later became sources for coastal recolonization; this scenario has been proposed to explain the genetic connectivity of *M*. *hispida* populations along the SWA coast [59] and may also apply to algal symbionts. Moreover, environmental characteristics particular to each island may help explain their higher symbiont diversity comparing to coastal locations. In our study, endosymbionts diversity in *F*. *gravida* was highest in coral samples from the RA. This oceanic island, the only atoll in the South Atlantic, is subject to daily tidal influence (0–3.8 m), whereby some pools become shallower at low tide with corals exposed to air, high temperatures and irradiance. Additionally, the atoll is comprised by two main pool habitats: open pools, which are permanently connected to the open ocean and more exposed to wave action; and closed pools, which remain isolated at low tide [60]. In the RA, higher diversity of endosymbionts was found in closed pools (*S*. *linucheae*, *Cladocopium* C1, *Cladocopium* C3, and *Fugacium* F3), whereas only *Cladocopium* C1 and *Cladocopium* C3 were found in open pools, suggesting that differences in physical environmental conditions (e.g., temperature and turbidity) help shape the association between *F*. *gravida* and their algal symbionts. Moreover, this is the first report on the occurrence of *Fugacium* sp. in the South Atlantic, which is not usually found in association with scleractinian corals. This endosymbiont genus is commonly found in foraminiferans hosts [61], although it is predominantly found in association with the scleractinian coral *Alveopora japonica* Eguchi, 1968 in temperate environments of the northwestern Pacific Ocean [62]. The physiological and ecological importance of *Fugacium* F3 for *F*. *gravida* remains unclear as it was found in only one colony mixed with *S*. *linucheae*. Besides, the large amount of differences between haplotypes of *Fugacium* F3 from this study and from the Pacific suggested that the lineage found in the SWA may be distinct than the others found in the Pacific. The other colony with mixed algal populations (*S*. *linucheae*/ *Cladocopium* C1) was also collected from the RA. The environmental variability described above may have contributed to this finding. Coral samples from the other SWA oceanic islands (FN and TR) harbored at least two different symbionts lineages, but *S*. *linucheae* was rare (< 10% of samples). Environmental factors may be the major determinants of symbiont diversity in FN and TR: both islands have a variety of habitats ranging from tide pools to 30 m deep reefs and FN is subjected to different wind and wave conditions depending on the period of the year and windward vs leeward sides of the island [63].

In conclusion, Symbiodiniaceae diversity associated with *F*. *gravida* was higher in the SWA compared to the TEA and MA. This coral host is generalist, which could be advantageous in case of a bleaching event, since different symbionts could assist the coral recovery. Additionally, distinct symbiotic algae (*S*. *linucheae*, *Cladocopium* C1 and *Cladocopium* C3) found in association with *F*. *gravida* are exposed to high temperatures and irradiance (tide pools) showing several endosymbionts resistant to harsh environmental conditions. Finally, the first occurrence of *Cladocopium* C130 and *Fugacium* F3 for the South Atlantic highlights the need for further studies in the region.

## Supporting information

S1 File Detailed description of the 12 collection sites of *Favia gravida* in the South Atlantic. (DOCX)
