# Correction: Biogeography of the endosymbiotic dinoflagellates (Symbiodiniaceae) community associated with the brooding coral *Favia gravida* in the Atlantic Ocean

**DOI:** 10.1371/journal.pone.0215167

**Published:** 2019-04-04

**Authors:** Mariana M. Teschima, Amana Garrido, Alexandra Paris, Flavia L. D. Nunes, Carla Zilberberg

There are errors in the Funding statement. The correct Funding statement is as follows: This study was financed in part by the Coordenação de Aperfeiçoamento de Pessoal de Nível Superior—Brasil (CAPES)—Finance Code 001 to MMT, Projeto Coral Vivo and its sponsors (Petrobras Socio-environmental Program and Arraial d'Ajuda Eco Parque) to MMT, SISBIOTA–Mar (CNPq 563276/2010-0 and FAPESC 6308/2011-8, PI: SR Floeter), and PELD/ILOC (CNPq 403740/2012-6, PI: CEL Ferreira). The funder had no role in study design, data collection and analysis, decision to publish, or preparation of the manuscript.
