# Supplementary material for: Biogeography of the endosymbiotic dinoflagellates (Symbiodiniaceae) community associated with the brooding coral Favia gravida in the Atlantic Ocean
Source: PLoS One. 2019 Mar 8;14(3):e0213519. doi: 10.1371/journal.pone.0213519 (PMC6407780; doi:10.1371/journal.pone.0213519)
Supplement: S1 File — (DOCX) [file pone.0213519.s001.docx]

**Supporting Information**

**S1 File. Collection sites description.** Detailed description of the 12 collection sites of *Favia gravida* in the South Atlantic.

1. Ceará (CE), southwestern Atlantic: coral colonies were collected at Praia do Aterro, a beach in the urban area of Fortaleza (population ca. 2,6 million), at the beginning of the dry season (July);
2. Rocas Atoll (RA), southwestern Atlantic oceanic island: this oceanic island, the only atoll in the South Atlantic, has an area of approximately 7 km² and is subjected to daily tidal influence, whereby some pools become shallower at low tide with corals exposed to air and high water temperatures ranging from 24‒36 °C [1]. The atoll is comprised by two main pool habitats, open pools, which are permanently connected to the open ocean and more exposed to wave action, and closed pools, which remain isolated at low tide [2]. Samples were collected at Piscina de Rocas (closed pool), Cemitério (closed pool), and Salão (open pool);
3. Fernando de Noronha Island (FN), southwestern Atlantic oceanic island: this island has a variety of habitats. Samples were collected from one tide pool (Baía dos Porcos) and one fringing reef up to 15 m depth (Laje Dois Irmãos). The western side of the island, where these reefs are located, is protected from the prevailing southeasterly winds that blow from April to November, resulting in low wave exposure. However, strong winds and higher swells are common in these reefs from November to March [3];
4. Rio Grande do Norte (RN), southwestern Atlantic: samples were collected from Rio do Fogo reefs, which are shallow patch reefs parallel to and ~7 km from the coast. This reef system is strongly influenced by terrestrial sediments [4];
5. Paraíba (PB), southwestern Atlantic: *F. gravida* colonies were collected at Picãozinho reefs, located ~1.5 km off the coast of João Pessoa (population ca. 800,000). Nearly 240 visitors may come daily by boat during peak tourist season, which can cause some disturbance in the form of trampling and pollution [5];
6. Pernambuco (PE), southwestern Atlantic: two sampling sites, Tamandaré and Serrambi, both affected by fresh water entering from nearby rivers and high sedimentation rates during the rainy season (January to June) [6];
7. Alagoas (AL), southwestern Atlantic: coral samples were collected in northern Alagoas (Ponta do Mangue) during the rainy season, which is characterized by higher turbidity. Coral reefs grow near the coastline and *F. gravida* are frequently found in tide pools or directly exposed to air during tide changes [7];
8. Porto Seguro (PS), southwestern Atlantic: located in southern Bahia, where sediment deposition on reefs, especially during winter storms, is strongly influenced by continental sources consisting of unconsolidated muddy sands and by a nearby river (Buranhém River) [4];
9. Abrolhos (AB), southwestern Atlantic: This is a coastal line of a group of five small islands with coral reefs that comprise the Abrolhos Brank reef complex off the southern coast of Bahia, it is the largest coral reef system in northeast Brazil. Samples were collected from Parcel das Paredes inner reefs ~10 km from the coast. This region is described as having sedimentation rates up to 10 mg cm^−^² day^−^¹ during winter, the maximum tolerable limit for healthy corals [8];
10. Trindade Island (TR), southwestern Atlantic oceanic island: located 1,100 km from the Brazilian coast, there are no true reefs and only three coral species occur [9]. Samples were collected from tide pools (Praia dos Andradas, Praia das Tartarugas, and Piscina Crista do Galo) and fringing reefs 6‒10 m deep (Noroeste, Lixo, Enseada do Monumento, Praia das Tartarugas, and Enseada dos Portugueses);
11. Ascension Island (ASC), mid-South Atlantic: located ~1,500 km from the African coast and ~2,300 km from the Brazilian coast, only three zooxanthellate scleractinian species are known from this oceanic island [10]. *Favia* *gravida* occurs mainly in tide pools and can be found in free-living form or attached to rocks [10,11]. Coral colonies were collected at Shelly Beach tide pools;
12. São Tomé Island (ST), Gulf of Guinea, Tropical Eastern Atlantic oceanic island: no true coral reefs occur along the west African coastline, however 11 scleractinian coral species are known from São Tomé [12]. Samples were collected in the main island of São Tomé and at the small islet of Rolas.

**References**

1. Fonseca AC, Villaça R, Knoppers B. Reef Flat Community Structure of Atol das Rocas, Northeast Brazil and Southwest Atlantic. J Mar Biol. 2012;2012: 1–10. doi:10.1155/2012/179128

2. Longo GO, Morais RA, Martins CDL, Mendes TC, Aued AW, Cândido D V., et al. Between-habitat variation of benthic cover, reef fish assemblage and feeding pressure on the benthos at the only atoll in South Atlantic: Rocas atoll, NE Brazil. PLoS One. 2015;10: 1–29. doi:10.1371/journal.pone.0127176

3. Krajewski JP, Floeter SR. Reef fish community structure of the Fernando de Noronha Archipelago (Equatorial Western Atlantic): The influence of exposure and benthic composition. Environ Biol Fishes. 2011;92: 25–40. doi:10.1007/s10641-011-9813-3

4. Leão ZMAN, Kikuchi RKP, Ferreira BP, Neves EG, Hilda H, Maida M, et al. Brazilian coral reefs in a period of global change: A synthesis. 2016;64: 97–116.

5. Costa RJ, Miranda GEC De. Análise dos estudos de capacidade de carga turística para os recifes de Picãozinho, Seixas (João Pessoa-PB) e Areia Vermelha (Cabedelo-PB). Cad Virtual Tur. 2016;16: 60–73.

6. Souza-Santos LP, Ribeiro VSS, Santos PJP, Fonseca-Genevois V. Seasonality of intertidal meiofauna on a tropical sandy beach in Tamandare Bay (Northeast Brazil). J Coast Res. 2003; 369–377.

7. Correia MD. Scleractinian corals (Cnidaria: Anthozoa) from reef ecosystems on the Alagoas coast, Brazil. J Mar Biol Assoc United Kingdom. 2010;91: 659–668. doi:10.1017/S0025315410000858

8. Segal B, Castro CB. Coral community structure and sedimentation at different distances. Ecologia. 2011;59: 119–129. doi:10.1590/S1679

9. Gasparini JL, Floeter SR. The shore fishes of Trindade Island, western South Atlantic. J Nat Hist. 2001;35: 1639–1656. doi:10.1080/002229301317092379

10. Zibrowius H, Wirtz P, Nunes FLD, Hoeksema BW, Benzoni F. Shallow-water scleractinian corals of Ascension Island, Central South Atlantic. J Mar Biol Assoc United Kingdom. 2014;97: 713–725. doi:10.1017/S0025315414001465

11. Hoeksema BW. Extreme morphological plasticity enables a free mode of life in *Favia gravida* at Ascension Island (South Atlantic). Mar Biodivers. 2012;42: 289–295. doi:10.1007/s12526-011-0106-z

12. Polidoro BA, Ralph GM, Strongin K, Harvey M, Carpenter KE, Arnold R, et al. The status of marine biodiversity in the Eastern Central Atlantic (West and Central Africa). Aquat Conserv Mar Freshw Ecosyst. 2017;27: 1021–1034. doi:10.1002/aqc.2744
